# Colonialism in the new digital health agenda

**DOI:** 10.1136/bmjgh-2023-014131

**Published:** 2024-02-27

**Authors:** Sharifah Sekalala, Tatenda Chatikobo

**Affiliations:** School of Law, University of Warwick, Coventry, UK

**Keywords:** Health policy, Health systems, Review, Health policies and all other topics, Public Health

## Abstract

The advancement of digital technologies has stimulated immense excitement about the possibilities of transforming healthcare, especially in resource-constrained contexts. For many, this rapid growth presents a ‘digital health revolution’. While this is true, there are also dangers that the proliferation of digital health in the global south reinforces existing colonialities. Underpinned by the rhetoric of modernity, rationality and progress, many countries in the global south are pushing for digital health transformation in ways that ignore robust regulation, increase commercialisation and disregard local contexts, which risks heightened inequalities. We propose a decolonial agenda for digital health which shifts the liner and simplistic understanding of digital innovation as the magic wand for health justice. In our proposed approach, we argue for both conceptual and empirical reimagination of digital health agendas in ways that centre indigenous and intersectional theories. This enables the prioritisation of local contexts and foregrounds digital health regulatory infrastructures as a possible site of both struggle and resistance. Our decolonial digital health agenda critically reflects on who is benefitting from digital health systems, centres communities and those with lived experiences and finally introduces robust regulation to counter the social harms of digitisation.

**Summary box**

- Neoliberal policies have led to digital health data being viewed as an asset for multinational corporations and philanthropic foundations in the global north, which we describe as a form of digital health coloniality.
- Although digital health initiatives are perceived to have the potential to transform health systems in the global south and act as an instrument for health justice, they often embed digital coloniality.
- Digital health initiatives entrench discriminatory border politics and racial hierarchies through software, hardware and storage and drastically increase the marketisation and commercialisation of health.
- A decolonial approach to understanding global health enables us to recognise how digital health coloniality affects the global south’s health outcomes and lived experiences and presents opportunities for reimagining digital health in ways that are restorative and transformative.
- Our decolonial agenda for digital health applies alternative decolonial lenses, such as indigenous African philosophical thought, that focus on centring community health experiences in developing robust and locally aligned digital health regulatory infrastructures as a mechanism of resistance to digital health coloniality.

## Introduction

The potential of digital health to transform health systems, especially in the global south, has been declared revolutionary.^1^ The COVID-19 pandemic further sped up the process of digital health, with countries scrambling to institute digital health surveillance, improve digital health systems through the promotion of telemedicine and provide public health information such as vaccinations through health apps.^2 3^ Postpandemic, digital health is increasingly being seen as critical for future pandemic preparedness, resilient health systems and making universal coverage possible.^4^ In August 2023, the WHO and the G20 India presidency announced a new global initiative on digital health to support the implementation of the Global Strategy on Digital Health 2020–2025.

Additionally, there has been significant interest and investment in artificial intelligence (AI) by powerful multinational corporations and philanthropic organisations predominantly in the global north for data-driven health solutions targeted mainly at global south countries. These corporations are also investing resources in infrastructure for health data storage, data-driven medical research and implementing predictive analytics for precision medicine.^5^ Datafication within health leads to a structural shift from social knowledge being a public asset towards a privately funded, processed and owned commodity.^6^ This creates a vicious circle where power and knowledge are concentrated in the hands of a few while inequalities are continuously entrenched, frustrating efforts to share benefits and promote health equity and justice.

In this article, we argue that while there is excitement about the digital health revolution and its potential to transform health systems in many countries of the global south, some of the risks are currently under-researched. This is due to an individualised examination of digital health systems that focuses mainly on individual rights, such as privacy and security, and a belief that removing barriers to access, skills and benefits would inevitably bridge digital divides and lead to equitable healthcare.^7^ In this article, we critically analyse how the rhetoric and practices of digitisation of health systems risk entrenching global health inequalities. This approach builds on an emerging literature that seeks to decolonise global health. This body of decolonial work critiques the ways in which knowledge and infrastructure from the global north are privileged in global health at the expense of those from the south.

## A review of global health coloniality

There is now an emerging body of literature on global health coloniality focusing on knowledge extraction and power asymmetries between the global north and south. The supremacy of specific knowledge systems manifests through the ways in which global health organisations operate, how decisions are made and how global health research and expertise are constructed and structured.^8^ Scholarship by researchers, scientists, institutions and publications predominantly in the global north is perceived to be superior and therefore the standard for informing global health decisions and practices everywhere.^9^ By design, this deprives the global south of spaces and opportunities to develop locally relevant knowledge systems independently, positioning predatory knowledge infrastructures as the only avenue for global health knowledge contribution. The ‘foreign gaze’ in global health is meant to exploit and extract knowledge resources from the global south as well as a mechanism for policing the boundary through political rhetoric that fuels and naturalises colonialist biases and harmful ideologies such as racist labelling to advance specific agendas, as witnessed during COVID-19.^9 10^ The coloniality of global health knowledge is also illustrated in the way grant funding for global health research is deployed and managed to privilege the global north institutions and researchers, who retain senior positions and decision-making powers in most global health institutions and collaborations, even for projects implemented in the global south.^11^ This intentionally removes knowledge reciprocity avenues and perpetuates a colonial system on the ways of being, becoming and doing in the South.^12 13^

Within the context of digital health, we pay particular attention to the way in which capitalism exacerbates these colonial logics. Within digital health, capital is currently concentrated in the hands of a few multinational technology corporations, largely based in the global north, who wield enormous influence on digital health research agendas, policies, infrastructures and technology deployments in a way that promotes and protects corporate interests. Corporations also exert and extend their influence over sociopolitical orders through philanthropic positioning as change agents for health development and humanitarian efforts.^14^ Through corporate social responsibility and private foundations, these enterprises often (re)frame digital health ideologies that align with and promote their corporate interests. This is evident in the way in which digital technologies are framed as the ‘magic prescription’ for all social, economic, political and environmental problems and obscure structural problems that afflict health systems.^14 15^

Due to their enormous grant-making capacity, philanthropists can prioritise certain health research and development in a way that pulls time and resources away from other significant health priorities.^16^ This leads to the reinforcement of colonial relations of dependency and the positioning of philanthropic organisations and their funders, who are mostly technology and financial corporations in the global north, as purveyors of global health and social knowledge everywhere with very little scrutiny and accountability. This influence has also permeated international organisations such as the WHO, where philanthropic organisations are using their financial clout to distort health priorities.^17^ Additionally, through the financing and support of private consultancy interventions that reproduce their ideologies at intergovernmental organisations, philanthropic organisations maintain decision-making powers at the highest structures of governance, often at the expense of member states and formalised stakeholders.^18^ These agenda-setting privileges result in health interventions that ignore deep-rooted structural socioeconomic factors and often result in failure.^19^

## Defining digital health coloniality

Digital coloniality refers to the systemic and structural violence of human life through technological systems and designs to exploit the everyday socialities, localities and temporalities of individuals. Digital coloniality benefits technological conglomerates and organisations situated predominantly in the global north.^20 21^ The premise of digital coloniality, centres on datafication, and is based on human life being a quantifiable commodity and raw material. In the context of global health, the ability to collect, process, store and use health data, therefore, becomes a form of power and violence through the reinforcement of existing hierarchies. Despite the universalising mission of digital health, it risks entrenching coloniality.

Underlying the digital health agenda is a reproduction of the colonial logic of modernity and rationality, which are characteristics of Eurocentricism. Digital health technologies are positioned to offer access to a preconceived and universalised idea of global health. Many governments are reproducing the modernity rhetoric in their anxieties around ‘not being left behind in the fourth industrial revolution’, ‘creating frictionless data transfers’, ‘our future is digital’, ‘the future of healthcare is Asia or Africa’ depending on which management consultant report you read or that ‘African digital natives’ are accelerating and driving the adoption of technologies.^22–25^ In digital health, modernity rhetoric serves the colonising spatial and temporal function of both dehumanising the global south as a place of otherness and non-being and placing the Western version of humanity and progress as something to aspire to everywhere.^26^ This is consistent with the colonial rearticulation of non-Western people in terms of deficiencies and as people without histories, futures, development or democracy who could only (re)gain their ontological density by adopting Western notions of being and becoming.^27 28^

Increasingly, multinational corporations, global institutional financers and philanthropic foundations are compelling governments in the global south to develop neoliberal policies and regulatory frameworks in ways that inevitably lead to the financialisation and assetisation of development, which also includes areas such as digital health.^15 29 30^ Given the global political economy of financing, digital health data becomes an asset that benefits institutions, foundations and corporations in the global north—at the expense of emerging global south ones. These foreign entities control and retain exclusive rights and private control over digital health assets such as health data, products and tools.^31^ Digital health actors in the global south face high barriers to entry, which reinforces market monopolies by global north institutions, foundations and corporations that have capital and therefore carte blanche in restricting, expanding and controlling the digital health market, which results in digital health data being owned as a private asset and not as a public good. This orientation creates power imbalances in favour of the global north institutions with capital, compromising the ability of public institutions, especially in the global south, to dispense obligations such as health access and other provisions, further entrenching inequalities and vulnerabilities.^31^

## The characteristics of digital health coloniality

### Border politics: closed to humans but open for digital health data

AI is perceived as having the potential to transform healthcare access globally, for example, by using machine learning to make predictions and personalise treatment strategies and using data models and algorithms to analyse medical scans and pathology images.^32^ As such, AI relies on massive, unrestricted flows of data and complex technological infrastructures to collect, store and analyse it. Corporations that train AI models for health are increasingly looking for electronic health records from all over the world, especially in the global south, where this data has been difficult to collate.^33^ For many in the global south, this involves the extraction of digital health data by corporations primarily located in the global north. The extraction of health data for AI is also facilitated by powerful international organisations, such as non-governmental organisations like the WHO and the United States Agency for International Development (USAID). These organisations are investing in massive electronic health record systems that will ultimately be used to develop global health data models.^34^ Underlying these systems is an ideal of data flowing freely across national borders. By contrast, the free movement of people from the global south to the global north is becoming even more difficult through stricter border controls.^35–37^

These border controls have a long history in global health, with countries in the global south historically being linked to vectors of disease. Using international law, countries from the global north attempted to stem the flow of citizens from the global south through international sanitary conferences from 1851 to 1938. When citizens of the global south were allowed passage, this was often contingent on strict quarantine rules.^38^ During previous pandemics such as Ebola, we saw the dual standards with which these border policies were applied, with citizens from the global south locked out while those in the global north were allowed free entry.^36^ The Omicron variant during the last COVID-19 pandemic brought this discrimination into sharp focus when many countries in the global north closed their borders to South Africa and other African countries after South Africa proactively sequenced the variant.^37^ Additionally, the continuing lack of human mobility continues today, with many global health scholars from the global south being refused entry to the global north for conferences and other international health collaborations.^35 39^

### Infrastructural colonialism through software, hardware and the cloud

Digital technology infrastructures, which include software, hardware and the cloud, are mainly dominated by a few conglomerates that are in the global north. Big tech corporations exert tech hegemony through racially extractive models of appropriation and exploitation of human life through data for economic gain. Digital health applications rely on an entire ecosystem that is protected through intellectual property rules.^40^ While there is a rhetoric of free software for technical developers such as Java, C++ and Python, many developers soon discover that they often need more sophisticated paid-for software in order to create sustainable applications. This is further compounded by the fact that digital application marketplaces such as the Apple App Store or the Google Play Store act as gatekeepers of code and often place software-related demands on developers who want to publish applications on their platforms.^41^ The process of application development also relies on increasingly powerful and expensive proprietary hardware in the form of computers, storage and graphics, which may be protected within the intellectual property (IP) system using copyright, trademarks and designs. Within AI, while free datasets have been met with great enthusiasm, which conveniently sidesteps that in the long term, many developers will still need to pay substantial amounts to access relevant AI models, which are all located in the global north.^42 43^ This is despite the fact that the AI models are trained on global data sets, many of which were acquired freely. Additionally, the use of secure servers for storage is restricted due to IP rights and proprietary information embedded in them, making it difficult for emerging global south companies to leverage these innovations for digital health application development. Thus, the promise of rhetoric that everyone can gain access to these digital tools and democratise digital health application development remains a fallacy, especially in global south contexts.

The privatisation of software and hardware is sustained through a global intellectual property system that allows companies in the global north exclusive rights of use over technologies. Under the 1995 Agreement on Trade-Related Aspects of Intellectual Property Rights, commonly known as TRIPS, inventors have exclusive rights to profit from their innovations for a period of at least 20 years. Digital health technologies usually include software and hardware. Patents can be used to protect the hardware in health-related systems such as sensors but some inventions relating to methods and protocols, such as software, may also be patentable if they are perceived as 'new and involve an inventive step'. This system of IP rights has created colonial legacies in access to healthcare.^44^ These colonial legacies have perhaps been best illustrated recently through the way IP control resulted in inequitable access to vaccines in the wake of the COVID-19 pandemic, with countries in the global north hoarding more vaccines than they needed while those in the global south struggled to secure them.^38 45^

Additionally, many countries in the global south depend on the global north for cloud services. With data centres located in the global north, the influence of big tech has filtered into global south government operations, some of which depend on external digital infrastructures such as cloud storage.^46^ Cloud computing can be described as ‘a model for enabling convenient, ubiquitous on-demand network access to a shared pool of computing resources.’^47^ The cloud has revolutionised storage and backup facilities and can be used to enable the sharing and accessibility of health data globally. Many of the earlier concerns about ‘the cloud’ were with security and privacy. However, the rise of end-to-end encryption has resolved some of the concerns, although global north countries such as the USA with its Cloud Act of 2018 can still request all USA cloud infrastructure providers to hand over data to law enforcement even if the location of data centres is abroad. The top cloud computing companies are still based in the global north, with the majority in the USA and Europe.^48^ Many countries are so dependent on this cloud infrastructure that even simple transfers between entities in the same country will often be reliant on a cloud that is often based in the global north.^49^ This means that even local companies in the global south using health apps for local purposes will be reliant on remote storage and backup facilities.

### Philanthropy, aid, marketisation and the distortion of public health agendas

With advancements in digital health innovation, thanks to big tech, funding instruments, capitalistic logic and market-driven dynamics are reshaping public health agendas.^50^ Venture capitalists fund start-up innovation with the sole purpose of making the highest returns possible and digital health has emerged as a potential field for commercialisation.^51^ This has meant that financial returns are prioritised over health gains, which can distort public health objectives and outcomes. Additionally, the increased focus on digital capital at a cost in the global south entrenches existing inequalities, stigmatisations and discriminations along intersectional lines and reinforces colonial relations of dependence and dominance.^52^

Private funding for public services has always revealed how certain agendas get prioritised over others.^53^ This approach to digital health funding disproportionately affects countries in the global south that might not have adequate resources to invest in digital health infrastructure for their national health agendas. Some of the global south’s digital health programmes are donor funded by international non-governmental organisations located in the global north.^15^ These organisations influence health outcomes and also participate in or are complicit in sustaining the cross-border migration of data. For example, most of the donor-funded projects, such as the MEASURE Evaluation’s Data for Impact project (D4I)—a USAID-funded project that works primarily in Sub-Saharan Africa to increase capacity to use data for improving health programmes^54^—do not explicitly state how they manage issues of data governance such as stewardship of data, location and mode of data storage and what sufficient safeguards exist for ensuring appropriate use of data.^55^ MEASURE Evaluation D4I is a USAID-funded project based at the Carolina Population Centre of the University of North Carolina that works in low and middle-income countries, mainly in Sub-Saharan Africa, to increase capacity and collect, analyse and use data for improving health programmes and policies.

Discussions and debates on issues of data governance remain fragmented and compartmentalised, possibly by design to maintain the current digital health data order.^55^ For example, corporations and organisations could still extract health data through secondary data infrastructures and mechanisms, therefore bypassing existing laws and regulations.

### Let us break things and the high cost of failure!

Mark Zuckerberg, the CEO of Meta, is quoted as saying, ‘Move fast and break things. Unless you are breaking stuff, you are not moving first enough.’^56^ While this may seem like the hyperbole of a billionaire, this mentality is endemic to digital innovation. Big tech companies, with their unlimited budgets, can afford to absorb smaller tech start-ups even with innovations that have not been developed and defined.^57^ This acquisition is premised not on making a profit out of the start-ups but rather on protecting their market dominance by monopolising the market and exterminating competitive products and services. Moreover, Microsoft and Alphabet are aggressively poaching tech talent from global south countries such as Kenya, Uganda, Nigeria and South Africa, leaving small tech start-ups understaffed, most of which cannot compete for the huge salary and benefit packages often threefold what is offered locally.^58^

While the African tech start-up ecosystem has been credited as one of the fastest-growing in the world, it has also seen a significant number of closures and shutdowns. This is due to the experimental nature of these tech start-up businesses and limited funding opportunities, which give an unfair advantage to the big tech conglomerates.^59^ For instance, the increased experimental approach to digital health that is endemic in digital health application developments in East Africa has detracted from efforts to realise universal health coverage and fragment the healthcare landscape in the region.^60^

## A decolonial agenda for digital health

We propose a decolonial agenda for digital health in which scholars and policymakers seek to recentre formerly colonised peoples from the global south in digital health plans. This agenda complements the existing body of global health scholarship that highlights possible resistance strategies to global health coloniality (table 1).

**Table 1:** Proposed approaches to restorative justice in global health.

Decentralising global health knowledge platforms	There are calls for global health knowledge platforms to be more inclusive, decentralised and centred on epistemic ecologies in the global south in terms of research and practice.^65–68^ This also includes using the insights from feminist, decolonial and critical scholarship on global health, digital technologies and political economics to inform digital health decisions.^69^ Such scholarship, some by global south scholars, has exposed the risks that digital technologies such as AI and LLMs could amplify, especially for people in marginalised contexts since such innovations are neither neutral nor objective. For example, a feminist reading of digital health inequalities would reveal how existing gender inequalities, which have been worsened by the socioeconomic fallout from the COVID-19 pandemic, intersect with harmful digital biases and gender disparities in digital health decision-making and tech designs to form extremely violent inequalities, which affect women with racial and ethnic minority backgrounds in profound ways.^70^ Similarly, critical research has revealed how data from crises is often incomplete, excluding those most affected. This paradoxically reproduces inequalities and makes the use of data in health emergencies potentially harmful.^14^
Accountability	There are calls for a shift in accountability, from seeing global health as a charity exercise to a mechanism for high-income countries to protect their interests. There are also calls for localised and bottom-up approaches to challenging health inequalities through policy development advocacy and building community-led health accountability-oriented social movements.^10 66 71–73^ Accountability also entails problematising the rhetoric that, given a chance, digital technologies such as AI and LLMs can enable a cost-effective, efficient and rapid approach to healthcare. Such rhetoric absolves governments of the responsibility to invest extensively in robust health infrastructures in spaces and places where they are needed the most.^15^
Strengthening mechanisms for resistance	New and existing social movements are essential for promoting global health equity and justice. While some limitations are being observed in the global health literature on health-related social movements, especially in marginalised contexts, health activism has the potential to play the decolonial function of dismantling hegemonic systems in global health.^74–76^ Approaches to resistance need to be attentive to and prepare for counter-resistance strategies from dominant interest groups aimed at ensuring that resistance campaigns in digital health are always reactive and articulate clearly what they see as desirable digital health developments.^76^
South-to-South partnership and solidarity	Global health practitioners and researchers, especially from the global south, need to use their experiences, influences and opportunities to (re)centre, work in solidarity and build alliances with those marginalised in global health.^19 77–79^ While recognising that strengthening individual control of data is important, this approach is not transformative enough to address digital health disparities. South-to-south solidarity could involve developing collective ownership and control of digital health data and data infrastructures for equitable benefit sharing and public good.^80^ Such a collective and decentralised orientation would ensure that those who contribute their data to the digital commons also have an opportunity in decision-making processes on data use.^81^

AI, artificial intelligence; LLM, language learning models.

**Figure 1:**
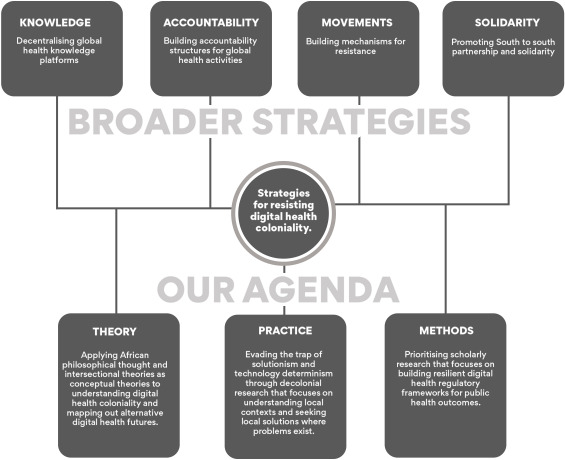
Consolidated resistance strategy for digital health coloniality.

Resisting digital health coloniality is a consolidated effort. This involves documented approaches such as democratising knowledge systems, developing structures for holding multinational corporations, governments, public institutions and philanthropic organisations to account, and building solid mechanisms for resistance and solidarity. Within digital health scholarship, we posit that we need to rethink these issues conceptually and empirically to consider alternative lenses, such as African philosophical thought and develop a more nuanced focus on digital health regulatory infrastructures (see figure 1 below).

Empirically, we will need to take the differences in national contexts seriously. The digitisation of health aims to flatten and provide uniform solutions across the globe. However, this is most certainly bound to fail, as the development and design of new technologies will need to centre on the needs of local communities. Conceptually, we need to think about what resistance to coloniality looks like in digital health. Critical scholarly work can also analyse the complexity and dialectical intermediation of global health coloniality and other colonialities and how this affects the nature, modes and structures of resistance. African philosophical thought and intersectional theories can be employed to do this. For example, the concept of Ubuntu, which encompasses humanistic ethics, accountability, conviviality, respect,^61^ could be reflexively used to look at the symbiotic and nuanced relationship of an individual to the community when it comes to health and how this can be used not only to critique the current digital health agendas but also inform the research, development and deployment of alternative digital health futures for the global south and by the global south. Ubuntu as a theory could inform the significance of collectivism and interdependence in some African settings and how it informs decision-making about one’s well-being as equally important as that of the community/spaces they live in. This contrasts with the current approach to digital health, which emphasises individualism and personal autonomy, for example, in terms of opting in and out of health applications. Consequently, concerns about privacy, security and harm are centred on an individualistic notion of being and existence and rarely consider the importance of community-mediated decision-making regarding health and technology use.

Second, we need to ask ourselves what we are trying to solve by using digital health technologies. There are real dangers of developing products that seek to solve problems that don’t exist, either through the transplantation of technologies and services or through limited and non-existent attention to local contexts. This creates fragmentation in health systems and exacerbate health disparities.

Lastly, and more importantly, we need proper regulation. For too long, there have been fears of regulation stifling innovation, but people’s health relies on robust regulatory frameworks that collectively centre health outcomes as opposed to providing products that are relevant for a small group of elites. This will entail well-designed empirical projects that seek to analyse the ways in which health apps are being utilised, the perceptions of users, and the impact on individuals and societies, both through traditional methods and also new ones such as legal epidemiology.

## Conclusion: three-point agenda for change

### From generalisations to empirical studies of national contexts

A recent commentary in the Lancet argued that with ‘the world’s largest burden of disease and the most severe shortage of healthcare workers’, African countries could benefit most from the proliferation of digital health solutions.^62^ Generalisations about multi-layered and multi-dimensional contexts appear regularly in both the mainstream press and in development documents. Continents like Africa are diverse, with myriad ranges of experiences. There is a need for empirical studies that appreciate this diversity and look at what works where and in what contexts.

### White elephants: do we need an app for this?

There is a replication of digital health applications ranging from payment to health insurance and telehealth services everywhere. During the pandemic, we have also seen the deployment of numerous COVID-19 applications redundantly in the same settings.^63 64^ In a typical colonialist fashion of divide and rule, these practices merely fragment and weaken existing health systems in places and times where they are needed the most. The approach of an app for anything further seeks to individualise health in global south settings, such as in Africa. We argue that the digitisation of health should be pursued to solve existing problems, not to look for problems.

### From digital sovereignty towards the pursuit of proper regulation

While digital sovereignty is seen as an approach to digital health regulation, in the global south, it might be more beneficial to have harmonised regulatory structures of resistance for pursuing health justice and solidarity. Applying theory-in-practice such as collectivism and conviviality, global south countries can work together in conceptualising and developing health data regulatory infrastructures that can be shared across nations. Structures and modes of resisting digital health coloniality can also be harmonised for a more effective and inclusive decolonial approach.

## Data Availability

There are no data in this work.
